# Adverse effects of salmeterol in asthma: a neuronal perspective

**DOI:** 10.1136/thx.2008.110916

**Published:** 2009-02-22

**Authors:** M Lommatzsch, Y Lindner, A Edner, K Bratke, M Kuepper, J C Virchow

**Affiliations:** Department of Pneumology, University of Rostock, Germany

## Abstract

**Background:**

Regular use of inhaled β_2_-agonists has been associated with a paradoxical loss of asthma control and a deterioration of airway hyper-responsiveness, but the underlying mechanism is unknown. The neurotrophin brain-derived neurotrophic factor (BDNF) has recently been identified as a mediator of airway hyper-responsiveness in asthma.

**Methods:**

Eighteen patients with mild allergic asthma who did not use any regular antiasthmatic therapy inhaled the long-acting β_2_-agonist salmeterol for 2 weeks followed by 2 weeks of combination therapy with salmeterol and the corticosteroid fluticasone. Airway responsiveness to histamine and BDNF concentrations in blood were assessed prior to entry, after 14 days of salmeterol therapy and after 14 days of combination therapy. In a separate experiment, salmeterol effects on BDNF release by human peripheral blood mononuclear cells were assessed.

**Results:**

Monotherapy with salmeterol significantly increased BDNF concentrations in serum and platelets. This increase was abolished by the addition of fluticasone to the treatment. The findings were confirmed in vitro: salmeterol increased the release of BDNF by mononuclear cells, and this was inhibited by co-incubation with fluticasone. Increased BDNF concentrations in serum and platelets correlated with the deterioration of airway hyper-responsiveness following salmeterol monotherapy. In contrast, there was no association between β_2_-receptor polymorphisms and changes in airway responsiveness.

**Conclusion:**

Increased BDNF concentrations may underly the adverse effects of salmeterol monotherapy on airway responsiveness in asthma.

**Trial registration number:**

NCT00736801.

Asthma is characterised by airway inflammation, airway hyper-responsiveness (AHR) and a reversible airflow limitation.^1^ Inhaled corticosteroids (ICSs) are the treatment of choice for asthma. In more severe asthma, international guidelines recommend that ICSs can be combined with inhaled long-acting β_2_-agonists (LABAs) such as salmeterol.^2^ Monotherapy with β_2_-agonists has not been recommended due to accumulating evidence suggesting a loss of control and an excess mortality in asthma with this treatment.^3^ Several studies reported that unbalanced use of short-acting sympathomimetic bronchodilators as well as LABAs can cause a deterioration in asthma control, and increase exacerbations and hospital admissions, most probably as a class effect of β_2_-agonists.^4^ ^5^ ^6^ ^7^ ^8^

Well-controlled clinical studies have demonstrated that regular inhalation of short acting β_2_-agonists such as fenoterol, albuterol and terbutaline increases airway responsiveness to histamine or methacholine.^4^ ^9^ ^10^ This effect is not attributable to a β_2_-receptor subsensitisation.^11^ ^12^ In addition, the regular use of albuterol has been shown to increase the allergen-induced early^13^ and late asthmatic response.^14^ Carefully conducted studies on the effect of regular use of LABAs in patients with asthma have only been performed in children where regular monotherapy with salmeterol also led to an increase in AHR.^15^ ^16^ Futhermore, regular inhalation of short- (terbutaline) as well as long-acting β_2_-agonists (salmeterol) led to a tolerance of the bronchoprotective actions of both drugs against non-specific bronchoconstrictor stimuli.^17^ ^18^ In two more recent large-scale trials, salmeterol treatment was even associated with excess mortality in asthma.^19^ ^20^ A trend towards excess mortality in asthma has recently also been reported for formoterol.^21^ However, the mechanism by which the regular inhalation of β_2_-agonists contributes to increased airway responsiveness and a loss in asthma control is unclear.

The neurotrophin brain-derived neurotrophic factor (BDNF), a crucial regulator of neuronal activity in adults,^22^ has been linked to several features of asthma. BDNF is upregulated in allergic airway inflammation and induces AHR and airway obstruction in an animal model of allergic asthma, via an increase of neuronal sensitivity and activity in the airways.^23^ ^24^ ^25^ ^26^ In patients with asthma, systemic concentrations of BDNF are increased and these concentrations correlate with AHR.^27^ Following local allergen challenge, endobronchial BDNF levels increase significantly in patients with asthma.^28^ In addition, there is evidence in human asthma that corticosteroids prevent allergen-induced increases in AHR^29^ and reduce BDNF concentrations.^27^ ^30^ ^31^ However, there is no information on the effects of β_2_-agonists on BDNF concentrations in asthma. In this report, we investigate the effect of monotherapy with a LABA on BDNF concentrations and airway responsiveness in patients with asthma.

## Methods

### Study design

**Figure 1:**
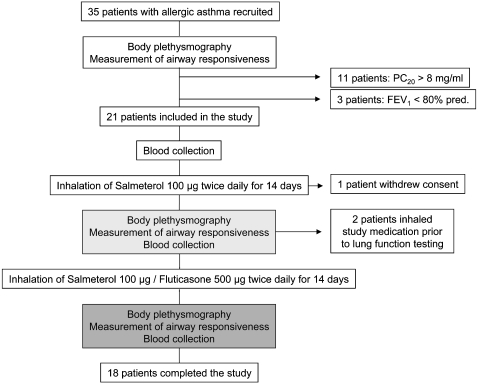
Study design. Body plethysmography, assessment of airway responsiveness to histamine and blood collection for brain-derived neurotrophic factor (BDNF) measurements were performed prior to entry (white box), after 14 days of salmeterol therapy (light grey box) and after 14 days of combination therapy (dark grey box). FEV_1_, forced expiratory volume in 1 s as a percentage of the predicted value (% pred.); PC_20_, provocative concentration of histamine causing a 20% fall in FEV_1_.

The study was performed between September and December 2006 in Rostock (Germany). Patients were recruited by newspaper advertisements. Patients were eligible when they met the following criteria: age >18 years, a doctor’s diagnosis of allergic asthma, a documented sensitisation to aero-allergens (pollen, animal hair or house dust mite), no regular treatment (only short-acting inhalers on demand were allowed), no history of or evidence for chronic disease other than asthma and no history of smoking. Prior to inclusion, recruited patients were assessed in the Department of Pneumology (University of Rostock, Germany). Recruited patients were included in the study if they met the following criteria: a prebronchodilator forced expiratory volume in 1 s (FEV_1_) >80% of the predicted value, a provocative concentration of histamine causing a 20% fall in FEV_1_ (PC_20_) of <8 mg histamine/ml and the absence of any signs or symptoms of an infection. After inclusion in the study, blood was collected and patients were instructed in the use of the inhalation device. Patients were asked to inhale salmeterol xinafoate 50 μg (Serevent Discus, GlaxoSmithKline (GSK), Brentford, Middlesex, UK) twice in the morning and twice in the evening for 2 weeks. In the following 2 weeks, patients were asked to inhale salmeterol xinafoate 50 μg and fluticasone propionate 250 μg (Viani Discus, GSK) twice in the morning and twice in the evening (fig 1). For safety reasons, patients were asked to record their peak flow daily, and to inform the monitor in case of any adverse event or symptomatic deterioration or a drop in peak expiratory flow (PEF) below 3 l/s. After 14 days of salmeterol therapy and after 14 days of combination therapy, body plethysmography, assessment of airway responsiveness and blood sampling were repeated (fig 1). The study medication was withheld for ⩾12 h prior to lung function testing. The study was approved by the ethics committee of the Ärztekammer Mecklenburg-Vorpommern (Rostock, Germany). Participating subjects gave their written informed consent.

### Clinical and laboratory procedures

Pulmonary function, airway responsiveness to histamine, blood cell counts and BDNF concentrations were assessed as previously described.27 32 Monocyte-enriched human peripheral blood mononuclear cells were isolated and cultured as described^27^ and stimulated with tumour necrosis factor α (TNFα, 50 ng/ml), in the presence or absence of salmeterol xinafoate and/or fluticasone propionate (GSK), for 24 h. Because fluticasone propionate was dissolved in alcohol, resulting in 0.01% alcohol in the culture, 0.01% alcohol was added to control and salmeterol xinafoate cultures. BDNF concentrations measured in supernatants were corrected for the percentage of non-viable cells to exclude artefacts due to corticosteroid-induced apoptosis, as described.^27^ Polymorphisms of the β_2_-receptor were analysed in blood containing ethylenediaminetetraacetic acid (EDTA) using PCR by a commercial laboratory (IMGM Laboratories, Martinsried, Germany).

### Statistical analysis

Data were analysed using SPSS (Chicago, Illinois, USA). Most parameters were non-normally distributed. Correlation analyses between the changes in BDNF concentrations and PC_20_ after 14 days of salmeterol therapy and after 14 days of combination therapy, and correlation analyses between β_2_-receptor polymorphisms and the changes of the PC_20_ after 14 days of salmeterol therapy were performed using the Spearman’s correlation coefficient. Lung function parameters, platelet counts and BDNF concentrations prior to entry, after 14 days of salmeterol therapy and after 14 days of combination therapy were compared using the signed ranks Wilcoxon test for related samples. Means of BDNF concentrations in cell culture supernatants after 24 h of incubation with TNFα alone and TNFα plus salmeterol xinafoate, fluticasone propionate or salmeterol xinafoate/fluticasone propionate were compared using analysis of variance (ANOVA; with SPSS). Probability values of p <0.05 were regarded as statistically significant.

## Results

### Patient characteristics

Thirty-five patients were recruited (fig 1). Of these, 14 patients did not meet the inclusion criteria (n = 11 had a PC_20_ of >8 mg/ml, and n = 3 had a prebronchodilator FEV_1_ <80% of the predicted value). Of the remaining 21 patients who were included in the study, 3 patients did not complete the study (one patient withdrew consent during salmeterol therapy due to an unacceptable subjective increase in asthma symptoms; two patients erroneously inhaled the study medication in the morning prior to lung function testing, and were excluded from the study). The baseline characteristics of the 18 patients which completed the study protocol (fig 1) are given in table 1. Three patients (16%) reported mild adverse effects (subjective discomfort or worsened dyspnoea) during salmeterol monotherapy, whereas none of the patients reported adverse effects during combination therapy. There were no severe adverse effects (leading to hospitalisation or death) during the study.

**Table 1:** Baseline patient characteristics

Patient no	Age	Sex	Years since diagnosis	PC_20_ (mg/ml)	FEV_1_ (% pred)	Allergies	β_2_-Receptor polymorphisms	
							arg16gly	gln27glu
1	20	M	13	4.0	91.9	P, D, A	Gly/Gly	Gln/Gln
2	19	M	5	3.8	81.8	D, A	Gly/Gly	Glu/Glu
3	20	M	8	4.5	105.7	P, D	Arg/Gly	Gln/Gln
4	18	M	5	0.2	84.2	P	Arg/Gly	Gln/Glu
5	36	F	5	5.0	103.6	P, D, A	Gly/Gly	Gln/Glu
6	19	F	11	3.3	102.2	P, D, A	Gly/Gly	Gln/Gln
7	39	F	6	4.2	86.8	P, A	Arg/Arg	Gln/Gln
8	20	F	10	3.8	109.1	P, D	Arg/Gly	Gln/Glu
9	42	F	10	5.5	115.3	P	Arg/Gly	Gln/Glu
10	44	M	8	0.4	82.5	D	Arg/Gly	Gln/Glu
11	18	M	3	2.8	95.8	P	Gly/Gly	Glu/Glu
12	29	M	3	5.5	88.7	P, A	Gly/Gly	Glu/Glu
13	18	M	6	0.8	89.9	D, A	Arg/Gly	Gln/Gln
14	38	M	28	0.8	92.0	P, D	Arg/Gly	Gln/Glu
15	26	F	10	1.5	93.2	P, D	Arg/Gly	Gln/Glu
16	19	M	10	1.4	97.3	P, D, A	Gly/Gly	Glu/Glu
17	19	F	8	8.0	87.0	P, D, A	Arg/Gly	Gln/Glu
18	20	F	15	2.7	106.0	P	Arg/Gly	Gln/Glu
**Median**	**20**		**8**	**3.6**	**92.6**			

A, animal hair; Arg, arginine; D, house dust mite; F, female; FEV_1_, forced expiratory volume in 1 s as a percentage of the predicted value (% pred); Gln, glutamine; Glu, glutamic acid; Gly, glycine; M, male; P, pollen; PC_20_, provocative concentration of histamine causing a 20% fall in FEV_1_.

### Lung function and AHR

**Figure 2:**
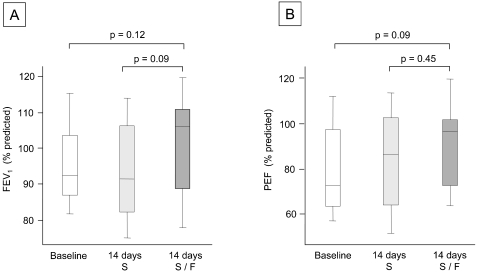
Lung function. Shown are forced expiratory volume in 1 s (FEV_1_; % predicted) values (A) and peak expiratory flow (PEF; % predicted) values (B) prior to entry (white box), after 14 days of salmeterol therapy (light grey box) and after 14 days of combination therapy (dark grey box) of n = 18 patients with allergic asthma. Boxplot graphs display the median (line within the box), interquartile range (edges of the box) and the range of all values less distant than 1.5 interquartile ranges from the upper or lower quartile (vertical lines). S, salmeterol; F, fluticasone.

**Figure 3:**
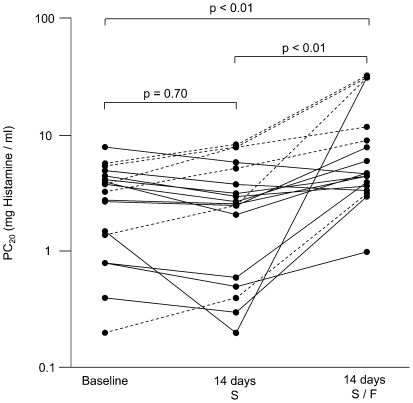
Airway responsiveness to histamine. PC_20_ (provocative concentration of histamine causing a 20% fall in the forced expiratory volume in 1 s) values are shown for each patient (n = 18 patients) prior to entry (Baseline), after 14 days of salmeterol therapy and after 14 days of combination therapy. Patients with a decrease in PC_20_ values after 14 days of salmeterol therapy are displayed with continuous lines. Patients with an increase in PC_20_ values after 14 days of salmeterol therapy are displayed with dashed lines. S, salmeterol; F, fluticasone.

The FEV_1_ (% predicted) and PEF (% predicted) did not change significantly following 14 days of salmeterol monotherapy (fig 2). Treatment with salmeterol and fluticasone led to a numerical increase in median FEV_1_ and PEF values, as compared with the baseline and with salmeterol monotherapy. However, the differences were not statistically significant (fig 2). Although there was no statistically significant change in AHR when the whole patient population was analysed, 12 of the 18 patients (67%) demonstrated an increase in AHR as measured by lower PC_20_ values to histamine following salmeterol monotherapy compared with baseline (fig 3). In contrast, when treated with a combination of salmeterol and fluticasone, nearly all patients displayed an increase in PC_20_ values (fig 3). AHR improved with combination therapy when compared with baseline in 15 patients (83%), and when compared with values obtained after salmeterol monotherapy in 16 patients (89%). This resulted in a statistically significant increase in the overall PC_20_ following the addition of fluticasone to salmeterol therapy (fig 3).

### BDNF concentrations in serum, platelets and plasma

**Figure 4:**
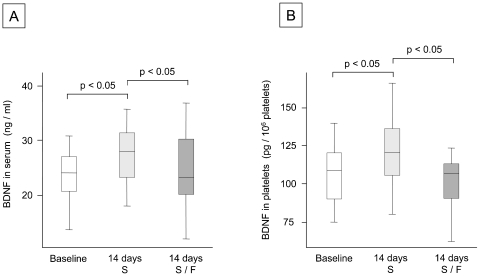
Brain-derived neurotrophic factor (BDNF) concentrations in serum and platelets. Shown are BDNF concentrations in serum (ng BDNF/ml serum) and platelets (pg BDNF/10^6^ platelets) prior to entry (white box), after 14 days of salmeterol monotherapy (light grey box) and after 14 days of combination therapy (dark grey box) of n = 18 patients with allergic asthma. Boxplot graphs display the median (line within the box), interquartile range (edges of the box) and the range of all values less distant than 1.5 interquartile ranges from the upper or lower quartile (vertical lines). S, salmeterol; F, fluticasone.

There was no statistically significant difference in BDNF concentrations in plasma at baseline (median: 0.09 ng/ml), after salmeterol monotherapy (median: 0.08 ng/ml) and after combination therapy (median: 0.08 ng/ml). In contrast, there was a statistically significant increase in BDNF concentrations in serum and platelets after salmeterol monotherapy compared with baseline. Both serum and platelet BDNF concentrations decreased significantly after 14 days of combination therapy (fig 4). There were no statistically significant changes in platelet counts at baseline (median: 232×10^9^/l), after salmeterol monotherapy (median: 247×10^9^/l) and after combination therapy (median: 255×10^9^/l).

### Association of BDNF with changes in PC_20_

**Figure 5:**
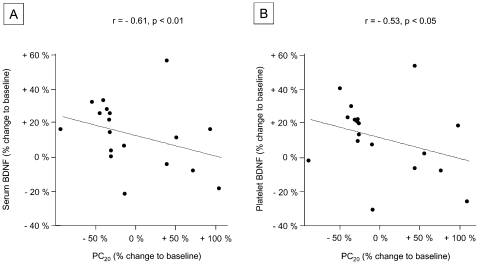
Association of brain-derived neurotrophic factor (BDNF) with changes in PC_20_ (provocative concentration of histamine causing a 20% fall in the forced expiratory volume in 1 s). Shown are correlations between changes in BDNF concentrations in serum (A) or platelets (B) and changes in the PC_20_ values (histamine) after 14 days of salmeterol therapy, as compared with the baseline before therapy. Each dot represents one patient; the line is the regression line calculated with SPSS (Chicago, Illinois, USA). Spearman’s rank correlation coefficient (r) and the significance of the correlation (p) are given above each graph.

Changes in BDNF concentrations in serum and platelets were correlated with the changes in PC_20_ values following salmeterol monotherapy (fig 5). Although BDNF levels decreased and PC_20_ values increased significantly following combination therapy with salmeterol and fluticasone, changes in BDNF levels were no longer correlated with the changes in PC_20_ following combination therapy (p>0.05 for serum and platelet BDNF concentrations).

### Effect of salmeterol on BDNF secretion by mononuclear cells

**Figure 6:**
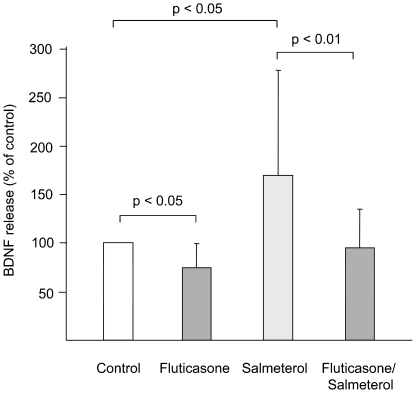
Brain-derived neurotrophic factor (BDNF) release by leucocytes in vitro. Monocyte-enriched human peripheral blood mononuclear cells of n = 22 healthy volunteers were stimulated with tumour necrosis factor α (TNFα, 50 ng/ml) for 24 h. Shown are BDNF concentrations (mean (SD)) in supernatants of wells containing fluticasone propionate (10^−7^ M), salmeterol xinafoate (10^−7^ M) and both fluticasone propionate (10^−7^ M) and salmeterol xinafoate (10^−7^ M), as compared with BDNF concentrations in the medium control.

To substantiate the in vivo effects of salmeterol and combination therapy on BDNF concentrations, monocyte-enriched peripheral blood mononuclear cells were isolated from 22 healthy volunteers, and stimulated with TNFα for 24 h. Fluticasone significantly suppressed BDNF secretion, whereas salmeterol significantly increased BDNF secretion, as compared with medium control (fig 6). BDNF secretion after co-incubation with salmeterol and fluticasone was not significantly different from medium control (p = 0.62), and significantly lower than BDNF secretion after incubation with salmeterol alone (fig 5).

### Impact of β_2_-receptor polymorphisms

In order to test if the effects of salmeterol on airway responsiveness were related to β_2_-receptor polymorphisms, the β_2_-receptor polymorphisms arg16gly (glycine for arginine in position 16) and gln27glu (glutamic acid for glutamine in position 27) were analysed (table 1). As far as the arg16gly polymorphism is concerned, 7 patients (39%) were glycine homozygotes, 1 patient (6%) was an arginine homozygote and 10 patients (55%) were heterozygotes. As far as the gln27glu polymorphism is concerned, 4 patients (22%) were glutamic acid homozygotes, 5 patients (28%) were glutamine homozygotes and 9 patients (50%) were heterozygotes (table 1). There was no statistically significant association between the changes in PC_20_ after salmeterol treatment and the β_2_-receptor polymorphism arg16gly (r = 0.11, p = 0.65) or the β_2_-receptor polymorphism gln27glu (r = 0.34, p = 0.17).

## Discussion

Inhaled β_2_-agonists provide rapid bronchodilation and have been shown to improve asthma control in a fixed combination with ICSs.^33^ ^34^ However, there is now increasing evidence for a paradoxical loss of asthma control and an increase in asthma mortality following β_2_-agonist monotherapy.^3^ ^19^ ^20^ An explanation for this observation is lacking. Several reports highlight that regular treatment with β_2_-agonists in patients with asthma can increase airway responsiveness^4^ ^9^ ^10^ due to mechanisms which are incompletely understood.^35^ There is, however, evidence that increases in airway responsiveness following β_2_-agonist treatment are at least in part explained by changes in the reactivity of airway nerves.^36^

Mediators with the potential to change neuronal reactivity in the airways are neurotrophins such as BDNF which induce long-term changes in neuronal function and activity.^22^ ^37^ Animal studies have shown that the production of BDNF by leucocytes and epithelia is strongly upregulated in allergic airway inflammation.^23^ ^26^ In a functional study, the inhibition of endogenous BDNF reduced AHR in allergen-challenged mice, whereas administration of recombinant BDNF was sufficient to induce AHR in healthy mice.^25^ In the same study, it was demonstrated that these effects of BDNF are due to changes in the neuronal reactivity within the airways.^25^ These findings have recently been confirmed in a study with guinea pigs.^38^ Thus, there is now accumulating evidence from animal models that BDNF enhances neuronal reactivity in the airways and contributes to neuronal dysfunction and AHR in allergic airway inflammation.^24^

In patients with asthma, enhanced local BDNF concentrations in the lung^28^ are mirrored by enhanced BDNF concentrations in circulating platelets.^27^ BDNF is produced neither by platelets nor by its precursors, but actively acquired by platelets.^39^ Therefore, platelet BDNF appears to be an estimate for the average BDNF secretion in organs of the human body over a period of several days.^32^ Accordingly, platelet BDNF (but not plasma BDNF) has been shown to correlate with the severity of AHR.^27^ This association may simply reflect the fact that enhanced BDNF concentrations in the airways lead to both AHR and an enhanced uptake of BDNF into circulating platelets. However, it is also conceivable that platelet BDNF plays a genuine role in asthma. Platelets have been shown to migrate actively into the lung and to contribute to functional changes within the airways in allergic airway inflammation.^40^ ^41^ Thus, it can be speculated that not only a BDNF overproduction in the airways by leucocytes and epithelia, but also an enhanced deposition of BDNF by platelets might contribute to the development of AHR in asthma.

In the present study, 14 days of treatment with salmeterol in patients with asthma led to a significant increase in platelet BDNF concentrations and this was correlated with changes in airway responsiveness. In addition, these effects were abolished by adding inhaled fluticasone to the treatment. Thus, the correlation between the changes in AHR and the changes in BDNF concentrations suggests that BDNF might indeed contribute to the development of AHR in asthma and to some of the adverse effects of a salmeterol monotherapy. The mechanisms underlying the induction of BDNF by salmeterol are as yet unclear. However, salmeterol has been shown to increase the transcription of genes with cAMP response elements in their promoters.^42^ Thus, as BDNF is known to have active cAMP response elements in its promoter,^43^ salmeterol could enhance BDNF transcription via this pathway.

Concomitant treatment with fluticasone led to a significant decrease in platelet BDNF levels in vivo and to a significant reduction in BDNF secretion by leucocytes in vitro. It has already been shown that ICSs such as fluticasone reduce BDNF secretion by leucocytes^27^ as well as BDNF serum levels in patients with allergic asthma.^31^ This is not specific for fluticasone because other corticosteroids (such as prednisolone and dexamethasone) also suppress BDNF secretion.^27^ ^30^ ICSs can prevent the increase in AHR following allergen challenge,^29^ which is associated with an increase in BDNF concentrations.^28^ Accordingly, the inhibition of BDNF production by ICSs as observed in this and previous studies^27^ further suggests that BDNF might indeed participate in the pathogenesis of AHR. Therefore, some of the beneficial effects of ICS in a combination with LABA in asthma might be related to a suppression of LABA-induced BDNF overexpression. It is of note that the decrease in BDNF levels did not correlate with the increase in PC_20_ values following combination therapy. This might be due to the fact that fluticasone not only suppresses BDNF, but has several other effects on a variety of cell types which influence airway responsiveness.

In this study, a deterioration in AHR following salmeterol monotherapy was observed in the majority (67%) of the patients. These data are consistent with studies showing adverse effects of long-acting^15^ ^16^ and short-acting β_2_-agonists^4^ ^9^ ^10^ on airway responsiveness in asthma. Genotyping for two β_2_-receptor polymorphisms implicated in the response to β_2_-agonists^44^ or persistence of asthma^45^ showed that the changes in PC_20_ values following salmeterol monotherapy were not related to these polymorphisms. Thus, although our study is clearly underpowered to exclude other detrimental effects of β_2_-receptor polymorphisms on asthma control, the observed effects on PC_20_ values do not appear to be related to these polymorphisms. This is in line with a recent analysis suggesting that β_2_-receptor polymorphisms do not affect the therapeutic response to LABA in patients with asthma.^46^ Our observation that the majority of patients with mild asthma developed an increase in AHR following salmeterol monotherapy suggests a susceptible subpopulation of patients which cannot be identified by β_2_-receptor polymorphisms or other clinical characteristics obtained in our study.

One limitation of this study is the lack of a crossover design. However, this was omitted due to ethical and safety concerns. A study arm in which patients would have left the study after regular monotherapy with a β_2_-agonist (with possibly detrimental effects on asthma control and mortality) was considered ethically inappropriate based on the strong recommendations in international guidelines that LABAs should not be used as monotherapy in asthma.^2^ Thus, although this may appear overcautious in patients with mild asthma, a crossover design was rejected. This decision has been confirmed in part by our results which showed a deterioration of AHR and mild adverse effects in a substantial portion of the participants following salmeterol monotherapy. Another potential pitfall in the design of our study is the lack of a placebo arm. However, although this might have at least theoretically improved the study, it was prospectively decided that this approach would not offer advantages because the high dose of the β_2_-agonist (100 μg of salmeterol twice daily) unblinds such a design due to its inherent side effects. Furthermore, in our patient population, baseline parameters are representative of a placebo arm since baseline treatment (short-acting bronchodilators on an as-needed basis) was maintained unchanged throughout the study.

In conclusion, we show that unbalanced monotherapy with salmeterol in patients with mild asthma increases BDNF production and storage and that changes in AHR are associated with this effect. We, therefore, hypothesise that augmented BDNF concentrations explain some of the adverse effects of β_2_-agonists in asthma. Further studies in patients with more severe airflow obstruction are warranted to confirm these findings.
